# Legume Flour or Bran: Sustainable, Fiber-Rich Ingredients for Extruded Snacks?

**DOI:** 10.3390/foods9111680

**Published:** 2020-11-17

**Authors:** Cristina Proserpio, Andrea Bresciani, Alessandra Marti, Ella Pagliarini

**Affiliations:** Department of Food, Environmental and Nutritional Sciences (DeFENS), Università degli Studi di Milano, 20133 Milan, Italy; andrea.bresciani@unimi.it (A.B.); alessandra.marti@unimi.it (A.M.); ella.pagliarini@unimi.it (E.P.)

**Keywords:** acceptance, sensory descriptive analysis, CATA, texture analyzer, pulses, green peas, chickpea, rice

## Abstract

The impact of using legume flour and bran on both sensory and texture properties in extruded, sustainable snack formulations was investigated. Sensory attributes determining consumer preference or rejection of legume-based snacks, as well as food neophobia and food technology neophobia were also explored. Seven samples of extruded snacks (R = 100% rice flour; C = 100% chickpea flour; P = 100% green pea flour; C30 = 30% chickpea bran and 70% rice flour; C15 = 15% chickpea bran and 85% rice flour; P30 = 30% green pea bran and 70% rice flour; P15 = 15% green pea bran and 85% rice flour) were subjected to the three-point bend method using a TA.XT plus texture analyzer. Seventy-two subjects (42 women; aged = 29.6 ± 9.3 years) evaluated the samples for liking and sensory properties by means of the check-all-that-apply (CATA) method. The sample made with 100% rice flour obtained the lowest liking scores, and it was not considered acceptable by the consumers. Samples P, C, C15, and P15 were the preferred ones. Crumbliness and mild flavor attributes positively influenced hedonic scores, whereas stickiness, dryness, hardness, and to a lesser extent, visual aspect affected them negatively. Neophilic and neutral subjects preferred the snacks compared with the neophobic ones, while no differences in liking scores were found regarding food technology neophobia. Extruded snacks with legume flour and bran were moderately accepted by consumers involved in the present study, albeit to a lesser extent for neophobic subjects, and could represent an interesting sustainable source of fiber and high-value proteins, as well as a valuable alternative to gluten-free foods present on the market.

## 1. Introduction

One of priorities of the food industry is to reduce the environmental impact of its production. This objective can be achieved using several strategies, including the improvement of food chains that have less of an impact than others and focusing on a “circular economy” to reintroduce bioactive components from waste or by-products into new food formulations. 

Legume production can satisfy both the abovementioned strategies. Firstly, a plant-based food system requires less resources in terms of water, land, and energy compared with a meat-based food system [1]. Legumes supply nitrogen for fertilization, since they can fix atmospheric nitrogen, thus reducing the amount of fertilizer used on the crops and increase proteins in animal feeding [2,3]. Secondly, milling by-products could be recovered to obtain bioactive components to be used as value-added ingredients in innovative food products. Among these components, legume bran has a high amount of dietary fiber, ranging from about 75% to 90% for chickpea and pea, respectively. Specifically, legume hull fiber is mostly insoluble fiber, whose purity is above the 80% [4].

It is well known that potential health benefits have been associated with the consumption of an appropriate amount of fiber [5]. Specifically, several epidemiological studies have highlighted that dietary fiber decreases the incidence of various diseases such as some types of cancers (e.g., colon and ovarian) [6,7], cardiovascular disease [8], and, in general, decreases the risk of mortality [9]. Moreover, it has been reported that the significant reduction in fiber consumption observed in industrialized countries is related to a worrying increase in cases of overweight people. Fiber intake in place of other macronutrients would lead to a decrease in calorie intake that could be extremely important for the overall health of the Western world [10]. However, the total dietary fiber consumed by the average individual is rather low, about <50%, of the recommended daily amount [11]. Even if proper nutrition requires legumes as a staple food in consumer diets, this recommendation is not often followed because cooking legumes can require a rather long preparation and because legumes are often not generally appreciated from the sensory point of view [12]. In recent years, a great deal of interest has been placed on partially replacing cereal-based products, such as pasta and bread, with legume flours to increase their nutritional profile [13].

Even if fiber-enrichment adds value in the eyes of the consumer [14], the addition of fiber in a food matrix causes changes in the production process, sensory properties, as well as texture and rheological parameters [15,16]. Therefore, the impact of adding fiber to a specific product needs to be studied. From a technological standpoint, fiber breaks down the starch–protein matrix, leading to important structural changes. Adding a small concentration of fiber could improve the structure of some products thanks to its ability to bind with water, but a high amount of fiber is almost always associated with a worsening of structural characteristics [17]. The types of fibers, as well as the food matrices in which these components are added, influence hardness, adhesiveness, and sensory attributes that cannot be generalized [18]. Previous findings have highlighted that pulse flours can be added up to of 40% (based on flour) in baked products without reducing their sensory quality [19], while other results have revealed that acceptability starts to decrease when more than 20% of wheat flour is replaced with that of legumes [20]. In addition to baked goods (e.g., bread and crackers), legumes might partially or totally replace cereals in the production of extruded snacks, products for which consumer demand is increasing since they can satisfy the demand for healthy, minimally processed, ready-to-eat foods.

Therefore, the aim of the present study was to investigate the impact of using legumes and legume bran on the sensory and texture properties of extruded snack formulations. The sensory attributes determining consumer preferences with regards to the experimental samples were studied. Food neophobia, which is the fear to try new and unfamiliar foods [21], and food technology neophobia, which refers to new food technologies [22], were also explored as behavioral attitudes playing a key role in defining consumer behavior.

## 2. Materials and Methods 

### 2.1. Materials

Flours from milled rice (82.00% carbohydrates; 9.13% proteins; 1.19% lipids; 0.95% fiber), decorticated green pea (59.00% carbohydrates; 25.00% protein; 1.97% lipids; 9.50% fiber), and decorticated chickpea (56.00% carbohydrates; 24.00% proteins; 6.60% lipids; 10.10% fiber) were kindly provided by Molino Peila S.p.A. (Valperga, Italy), as well as the bran obtained from green pea (92.00% fiber; 3.30% proteins; 0.22% lipids) and chickpea (78.00% fiber; 11.20% proteins; 5.30% lipids). All values are expressed on dry basis.

Co-extruded snacks were prepared from rice (R), green pea (P), and chickpea (C). Moreover, bran from both green pea and chickpea were included in rice-based snack formulation at 15% and 30% levels, obtaining four different bran-enriched samples: C15, C30, P15, and P30. Overall, seven formulations were tested. Co-extruded snacks were produced at an industrial level by Fudex Group S.p.A. (Settimo Torinese, Italy) in the shape of bars. Extrusion was performed using a co-rotating twin-screw extruder (model 2FB90; screw speed: 150 rpm; temperature: 110 °C; pressure: 70 bar; Settimo Torinese, Italy). 

### 2.2. Instrumental Texture Analysis 

The textural properties of the snacks were determined by a three-point bend method using a TA.XT plus texture analyzer (Stable Micro Systems Ltd., Godalming, UK) equipped with a 100 N load cell. Snack bars were compressed with the Heavy, Duty Platform/Three Point Bending (HDP/3PB) probe at a crosshead speed of 1 mm/s to 5 mm of the original diameter of the snack. The compression generated a curve with the force over distance. The highest value of force was taken as a measurement for hardness. The test was carried out on 35 pieces for each sample, and the average value was considered.

### 2.3. Sensory Evaluations

#### 2.3.1. Subjects

Seventy-two subjects (42 women; mean age: 29.6 ± 9.3 years) were recruited among students and employees of the Faculty of Agriculture and Food Sciences of the University of Milan. The exclusion criteria were as follows: subjects who did not like rice and legumes, subjects suffering from food intolerances and allergies, as well as those who were on medical treatments that could modify taste perception. This study, approved by the Ethics Committee of the University of Milan, was conducted in compliance with the principles laid down in the Declaration of Helsinki. All subjects provided informed, written consent prior to participation.

#### 2.3.2. Hedonic Evaluation

Subjects were asked to taste the products and to express their liking using a labeled affective magnitude (LAM) scale, anchored by the extremes “greatest imaginable dislike” (score 0) and “greatest imaginable like” (score 100) [23]. Prior to tasting, the experimenters provided to the participants instructions for the use of the scale.

#### 2.3.3. Sensory Descriptive Evaluation

A separate group of 12 untrained subjects (mean age: 22.0 ± 4.1 years) were involved in a focus group, wherein they used a free listing questionnaire to define the appropriate sensory attributes to describe the extruded snacks [24]. Subjects had to evaluate the sensory characteristics of the snacks and identify all attributes for describing their color, appearance, odor, taste, flavor, and texture. After the development of the individual lexicon, an open discussion was made, and sensory attributes were selected by the experimenters considering the most commonly mentioned (frequency of terms selection at least of 40%) words in order to avoid synonyms [25]. Finally, the check-all-that-apply questionnaire consisted of a list of 23 sensory attributes: 3 for the appearance (dark yellow, light yellow, and green), 6 for the odor (strong, mild, toasted, rice, whole-meal, and legume), 3 for the taste (sweet, bitter, and salty), 6 for the flavor (strong, mild, rice, peas, chickpeas, and spicy), and 5 for the texture (crumbly, sticky, hard, porous, and dry). Subjects were asked to select the terms best describing each sample. Attributes’ positions were randomized using the “to assessor” list order allocation scheme [26].

#### 2.3.4. Questionnaires 

##### Food Neophobia Scale

Neophobic traits were investigated through the Food Neophobia Scale (FNS) developed by Pliner and Hobden (1992) [21]. The FNS consists of 10 statements each offering 7 graded response alternatives, from “strongly disagree” (score 1) to “strongly agree” (score 7). After reversing the negatively worded statements, the FNS score was calculated as a sum of the responses, yielding a range of 10–70. 

##### Food Technology Neophobia Scale

In order to investigate individual attitudes toward new food technologies, the Food Technology Neophobia Scale (FTNS) [22], consisting of 13 items, was used. Each statement offers 7 graded alternative responses, from “strongly disagree” (score 1) to “strongly agree” (score 7). Four of the 13 items reflect food neophilia, so responses had to be reversed in order to calculate the final neophobia score. The FTNS score was calculated as a sum of the participant’s answers for each statement, yielding a range from 13 to 91. Higher scores indicate a higher food technology neophobia level. 

#### 2.3.5. Experimental Procedure

Subjects attended one online session and one laboratory session. During the online session, they were asked to complete a questionnaire including demographic variables and the food neophobia and the food technology neophobia scales. Subsequently, they were invited at the sensory and consumer science laboratory designed according to ISO guidelines (ISO 8589 2007) and were asked to refrain from consuming anything but water for 2 h before the test. 

Samples were provided to the participants following a monadic presentation (one at a time) in a serving portion of approximately 30 g. The experimental samples were presented to the participants in plastic plates labeled with three-digit codes. Water was available for rinsing the palate between the samples. For each sample, subjects had to evaluate their overall liking and perform a sensory descriptive analysis by means of the check-all-that-apply (CATA) methodology. The entire session took approximately 30 min. Data were collected using the Fizz v2.47 software program (Biosystemes; Couternon, France).

### 2.4. Data Analysis

One-way analysis of variance (ANOVA) was applied to the data obtained from instrumental texture analysis, and the least significant differences were calculated by the Tukey’s HSD test.

ANOVA model was performed on overall liking scores considering samples (R, P, C, C15, C30, P15, and P30), gender (women and men), age (≤26 years old; >26 years old) and their interactions as factors. When a significant difference (*p* < 0.05) was found, the LSD post hoc test was performed as a multiple comparison test. 

The frequency of mention for each term of the CATA questionnaire was determined by counting the number of consumers who used that term to describe each sample. Cochran’s *Q* test was applied to identify which sensory attributes were discriminating among samples. The relationship between samples and sensory attributes was evaluated by means of correspondence analysis (CA). The influence of sensory attributes’ perception on hedonic scores was also investigated by means of penalty-lift analysis. This analysis suggests which sensory attributes are significantly (*p* < 0.05) positively or negatively associated with hedonic responses [27].

Correlations between instrumental and sensory texture data were examined using Pearson’s correlation coefficient with a minimum significance level defined as *p* < 0.05.

The internal consistency reliability of the food technology and food technology neophobia scale was explored by Cronbach’s alpha. ANOVAs were performed on FTNS and FNS scores considering age, gender, and their interactions as factors. To investigate the relationship between food neophobic traits and snack liking, subjects were categorized according to their neophobia scores into the following three groups: adults with scores in the lower 25th percentile of FNS scores, score <14 (Neophilic_FNS); adults with scores between the 25th and 75th percentiles, 14 ≤ FNS score ≤ 31 (Neutral_FNS); and adults with scores >31 (Neophobic_FNS). The same approach was used to identify subjects showing a lower (score < 31; Neophilic_FTNS), medium (31 ≤ FNTS score ≤ 46; Neutral_FTNS), or higher (score > 46; Neophobic_FTNS) level of food technology neophobia. 

ANOVA models were performed on liking data considering FNS level, FTNS level, gender, age, and their interactions as factors. All analyses were performed using IBM SPSS Statistics for Windows, Version 24.0 (IBM Corp., Armonk, NY, USA) and XLSTAT (Version 2019.2.2, Addinsoft™, Boston, MA, USA). 

## 3. Results

### 3.1. Hardness

Snack hardness is shown in Table 1. Snacks based on chickpea showed the highest value, almost two-fold higher than that of rice snack, which was used as control. On the other hand, snacks from green pea showed the least resistance to breakage. The addition of 15% bran from either green pea or chickpea did not significantly affect the snack texture in comparison with the 100% rice snack. Conversely, the type of bran was relevant when the milling by-product was included at 30% level. Specifically, adding 30% chickpea bran significantly decreased the force necessary to break the snack. On the contrary, in the case of 30% green pea bran, an increase (although not significant) in hardness values was recorded.

### 3.2. Hedonic Evaluation

Hedonic evaluation results are provided in Table 2. A significant sample effect was found for liking scores. The rice sample obtained the lowest liking score and was not considered acceptable by the consumers (mean hedonic score lower than middle of the scale = 50), while the samples with 100% pea and C15 were the preferred. Comparable liking scores were also provided for samples made with 100% C as well as formulations with 15% pea bran. These two last formulations were in turn comparable to the snacks made with legume bran at 30% (C30 and P30). 

A significant gender effect on liking scores was also found, with men providing generally higher scores compared to women. Moreover, younger subjects gave generally higher scores compared to older subjects. The two- and three-way interactions were not significant.

### 3.3. Sensory Descriptive Evaluation

The frequency table of terms checked by consumers to describe snack samples is reported in Table 3.

Cochran’s Q test yielded both discriminating and non-discriminating sensory attributes. Significant differences were found in the frequency of mention for 19 out of 23 terms for the five categories considered, suggesting that consumers perceived differences between samples in terms of their sensory characteristics. The sensory attributes that were not useful in order to discriminate samples were: mild odor, salty taste, bitter taste, and dry. In fact, snacks samples were generally characterized by a mild odor and low salty and bitter tastes.

**Figure 1:**
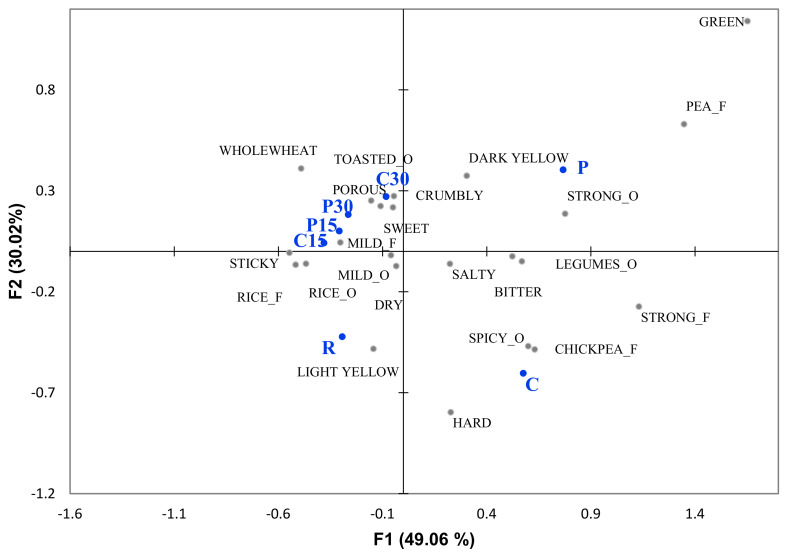
Correspondence analysis from check-all-that-apply data. Snack samples are reported in blue; the sensory attributes in black (O = odor, F = flavor).

A bi-plot of the products based on sensory descriptive analysis was obtained by means of a correspondence analysis (CA). The CA performed on the total frequency of participants’ counts for each attribute resulted in two dimensions accounting for 79.09% of variance of data. As shown in Figure 1, samples were discriminated according to bran percentages, with all samples containing bran (C15, C30, P15, and P30) positioned in the upper left side of the map well separated from the other samples not containing bran. In the other three quadrants, sample with 100% legumes (C and P) and 100% rice (R) were positioned.

**Figure 2:**
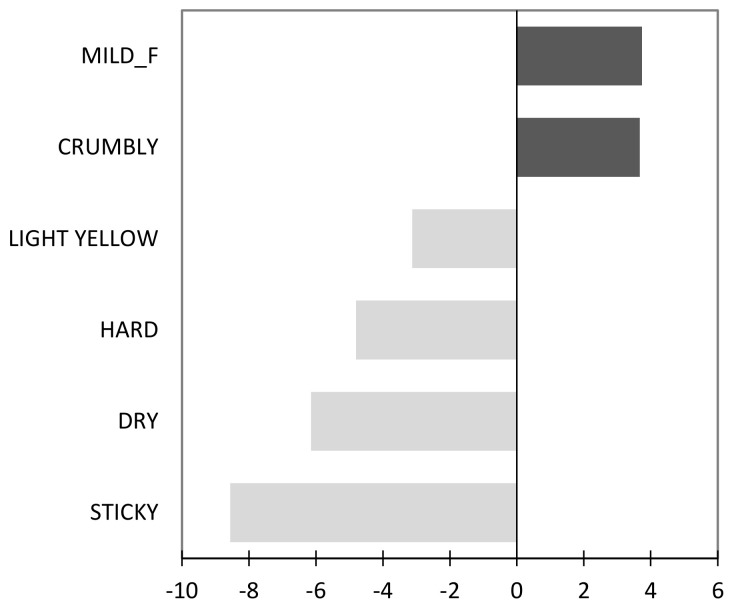
Penalty-lift analysis of sensory attributes across all snack samples. Only attributes that resulted in significant increase or decrease in overall liking are presented. (F = flavor).

The main sensory attributes that significantly (*p* < 0.05) influenced consumer hedonic perception are reported in Figure 2. Penalty analysis results revealed that two sensory attributes played a positive influence (drivers of liking: mild flavor and crumbly), and five attributes had a negative influence (drivers of disliking: light-yellow color, hard, dry, and sticky).

Looking to Pearson’s correlation coefficient in Table 4, significant correlation was found between texture results obtained by instrumental measurement and sensory data. In particular, positive correlations (*p* = 0.07) were highlighted between hardness (N) and “hard” attribute, while significant negative correlations were found between hardness (N) and crumbly, porous attributes. 

### 3.4. Food Neophobia 

**Figure 3:**
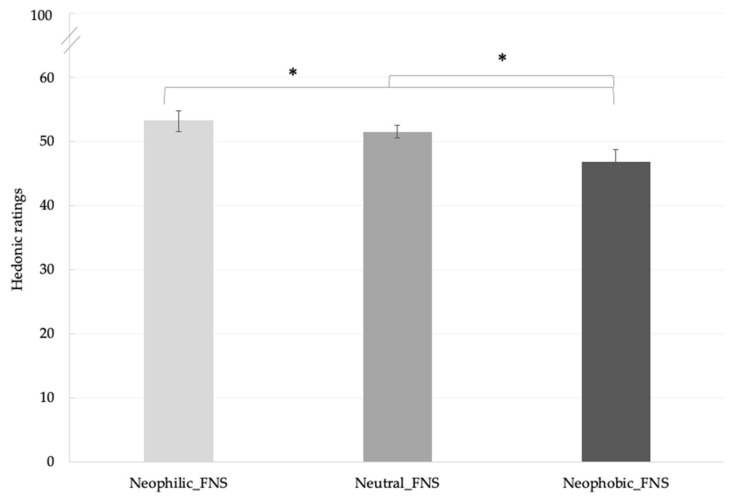
Mean liking scores ± SEM according to food neophobia levels. * *p* < 0.05. FNS = Food Neophobia Scale.

Satisfactory internal consistency of food neophobia scale, as calculated by Cronbach’s alpha test (Cronbach’s alpha = 0.92), was observed among items. The mean food neophobia value of subjects involved was 23.4 ± 12.5. No significant differences in neophobic traits could be attributed to gender and age (F_(1,68)_ = 0.44, *p* = 0.51; F_(1,68)_ = 1.22, *p* = 0.27, respectively). A significant effect of food neophobia on liking scores was found (F_(2420)_ = 3.46, *p* = 0.03). As reported in Figure 3, neophilic and neutral subjects gave generally significant higher liking scores (53.2 ± 1.6; 51.5 ± 0.9, respectively) compared with neophobic subjects (46.8 ± 1.9). 

**Figure 4:**
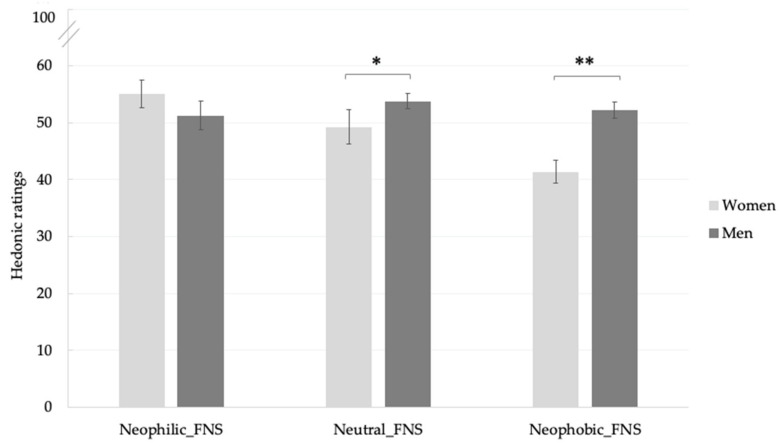
Mean liking scores ± SEM according to food neophobia levels and gender. * *p* < 0.05; ** *p* < 0.01. FNS = Food Neophobia Scale.

The food neophobia level × gender interaction was also significant (F_(2420)_ = 4.58, *p* = 0.01). As reported in Figure 4, no significant differences were found in hedonic scores according to gender in neophilic subjects (women: 55.1 ± 2.0; men 51.3 ± 2.5), while significant higher scores were provided by men with neutral food neophobia level (53.8 ± 1.4) and neophobic FNS (52.2 ± 3.0) compared with women (Neutral_FNS: 49.2 ± 1.3; Neophobic_FNS: 41.1 ± 2.4). No significant food neophobia level × sample effect was found.

### 3.5. Food Technology Neophobia 

Cronbach’s alpha for the 13 items in the FTNS assessment showed a satisfactory internal consistency (Cronbach’s alpha = 0.82). The mean food technology neophobia value was 40.3 ± 1.3. Significant differences (F_(1,68)_ = 4.42, *p* = 0.03) in neophobic traits according to age were found, with higher scores provided by subjects >26 years old (42.9 ± 1.7) compared with younger subjects (37.5 ± 1.9). No gender and gender × age effects were found on FTNS scores (F_(1,68)_ = 0.22, *p* = 0.64; F_(1,68)_ = 0.62, *p* = 0.43, respectively). 

As regards the influence of food technology neophobia level on liking scores, no effect was found (F_(1420)_ = 0.72, *p* = 0.48)

## 4. Discussion

In the present study, the use of chickpea and green pea flour and related bran in extruded snack formulations was investigated considering both sensory and texture properties. Sensory attributes influencing consumer preferences were characterized. Moreover, food neophobia and food technology neophobia were considered to define whether these behavioral attitudes could impact on hedonic perception.

Even though the use of legumes as a high-fiber and high-protein ingredient in food formulation has been widely investigated [28], to our knowledge this is one of the first studies that has evaluated consumer responses to extruded snacks containing different percentages of chickpea and green pea bran as sustainable food ingredients.

Samples developed with 100% chickpea and green pea, as well as samples with different percentages of legume bran, obtained significantly higher liking scores compared with the control sample made only with rice. These results suggest that the legume-based formulations developed here have a better market potential compared with the more traditional rice-based snacks. Legume-based snacks represent a promising gluten-free alternative not only for subjects with gluten allergy or intolerance but also for those who follow gluten-restricted diets for health reasons [29]. A gluten-free diet is actually one of the most popular diets, with a greater number of people avoiding gluten for nonmedical reasons than those who are dealing with a gluten-related disorder [30]. Moreover, due to their high-fiber content, the consumption of the legume-based snacks could help consumers reach their daily recommended intake of dietary fiber, which could have a potentially positive health effect. Indeed, despite the proven beneficial effects associated with a fiber-rich diet, the average intake of such components in adults is lower than the recommended daily intake [11]. In this context, food products, such as minimally processed snacks and ready-to-eat foods, with a low fat and salt, high fiber, and high-value proteins could be part of a balanced diet and lead to a consequent good health status [31]. Cereal-based snacks are mainly produced by extrusion-cooking, i.e., a relatively cheap, easy, and versatile technology that allows the production of a variety of textures and shapes that appeal to consumers [32]. The positive effects of extrusion on nutritional traits, including the decrease in antinutritional factors and the increase in soluble dietary fiber and in protein and starch digestibility, have been widely discussed [32,33,34]. On the other hand, extrusion might cause the loss of heat-labile vitamins and the reduction of the nutritional value of proteins, due to the Maillard reaction between protein and sugars.

The results reported in this paper agreed with the study of Balasubramanian and collaborators (2012) [35] who found that extruded samples made with black gram, green gram, lentil, and peas were well accepted. However, since previous sensory data on extruded snacks with legumes were obtained involving a small number of consumers, and thus not leading to robust and reliable results, the comparison between our hedonic data and previous results is not indicative. Generally, the replacement of cereals with legumes leads to a general trend toward decrease in food acceptability as the percentage of legumes increases [36]; however, it greatly depends on the food matrices used and how the process conditions are adapted with respect to the change in formulation. Indeed, legume flour in some products, such as biscuits and pasta, can enhance food acceptability [37].

It should also be pointed out that encouraging the legume chain represents an important sustainable action that can help to reduce greenhouse gas emissions, break the cycle of pests and diseases with crop diversification in agroecosystems, and contribute to protein production [38]. Moreover, using legume bran as a value-added ingredient for new food formulations reduces the environmental impact of this food chain [39]. Descriptive analyses revealed that some sensory evaluations were more affected by the type of legumes used in snack formulations rather than by the quantity of bran added. This is in line with evidence that changes introduced by the addition of bran are much more significant in wheat flour products than in gluten-free products that do not have such a complex and functional matrix as a gluten network has. To corroborate this hypothesis, it has been reported that fiber addition generally reduces acceptability in terms of consistency, flavor, and appearance, although when initial acceptability is low, as for gluten-free products, fiber addition can improve consumer preference [40,41,42].

Among sensory attributes, texture was found to be the most interesting. Indeed, sensory descriptive analysis revealed that, besides mild flavor, the other sensory attributes that positively affected overall hedonic responses were related to texture properties. Although texture has been referred to as the “forgotten attribute” due to the little attention it has received for several years [43], it is a complex sensory dimension including tactile, visual, and auditory sensations, playing an important role in defining consumer responses [44]. The present findings indicated that crumbliness of the products was an important driver of consumer preference. Interestingly, three out of four attributes responsible for the negative scores were related to texture. Our results are in line with previous research that found texture to be a critical factor for consumer acceptance of many kinds of food products [45].

In accordance with the sensory data, instrumental data showed chickpea snacks to have the greatest hardness, whereas green pea products were found to be less hard. Sensory and instrumental texture parameters were related to each other with the term “hard” being the most often mentioned when describing the chickpea sample. Differences in chemical composition might account for differences in texture. Specifically, the higher lipid content in chickpea might have favored amylose–lipid complex formation during extrusion, thus limiting starch swelling and gelatinization and accounting for a firmer structure. The addition of different percentages of legume bran led to an increase in hardness values. These results are corroborated by evidence reporting that the integration of fiber- and protein-rich plant by-products generally results in dense, hard extrudates due to several factors. Apart from starch dilution, fiber can interrupt the starch matrix and disrupt the bubble cells, leading to poorer texture (i.e., great hardness) [34]. Moreover, both proteins and fiber may compete with starch for free water, thus decreasing the occurrence of starch gelatinization. Our results suggest that reformulating snacks with 15% of legume bran will have no effect in limiting starch gelatinization, leading to products with textural features similar to those of rice snacks.

The type of bran seems to play a role only at high enrichment levels (i.e., 30%), with green pea bran and chickpea bran impacting the snack texture in an opposite way. Pea bran—being higher in fiber—could absorb water during processing, limiting its availability for starch gelatinization, thus resulting in a more compact structure with high hardness values. Besides differences in the chemical composition and, eventually, in the structural and functional characteristics of fiber, interactions between rice starch and legume fiber might also be considered.

Although the impact of either legume or plant-food processing by-products has been investigated [34,46], a direct comparison of our data with those found by other researchers is difficult. Indeed, snack features depend on several factors including moisture content, temperature, screw speed, die dimension, and screw profile. Adjusting such processing parameters would enable the creation of a wide variety of extruded food products with different structure–texture properties. Specifically, the snack produced in the present study is a co-extruded snack characterized by an extrusion-cooked outer shell that is later filled with either a savory or sweet filling. To the best of our knowledge, there are no studies dealing with the textural features (measured by instrumental analysis) of this kind of product.

Moving on to the behavioral attitudes that could have a role in the acceptance of high-fiber products, food neophobia data indicated that subjects scoring low to medium for neophobia gave higher liking scores to all samples compared to subjects scoring high. Generally, it is widely reported that neophobic subjects prefer less vegetable-based foods, with high fiber amount, compared with neophilic ones [47]. The present data are in line with a previous finding that demonstrated—in a large sample of children from five different European countries—that subjects more prone to try and eat new/unfamiliar food appreciated more experimental samples enriched in fiber [48]. Accordingly, it is well established that food-neophobic subjects are diffident in trying and buying novel foods, while neophilic ones tend to have a wide and varied diet [49]. No food technology neophobia effect was found regarding sample acceptability, while recent findings showed that most adolescents with a low level of food technology neophobia appreciated a flat bread with mushroom powder rich in ß-glucans compared with a control sample containing only wheat flour [50]. These contrasting results could be associated with the consumer sample involved in the study. Indeed, in the present research, people with knowledge about food science and technology were recruited as subjects, and this should be mentioned as a limitation of the study, whereas the adolescents involved in the previous mentioned research were more naïve consumers. 

## 5. Conclusions

Snack products with legume flour and bran represent an interesting food formulation for two reasons. From a nutritional standpoint, these products incorporating milling by-products at a high percentage represent an interesting source of fiber, as well as a valuable food alternative in the worldwide increasing demand for high-fiber and gluten-free products. From a sustainable point of view, the exploitation of milling by-products could reduce the environmental impact of this food category. All legume-based products containing bran in our study were accepted by the consumers involved, even if the hedonic scores were rather low. Crumbliness and mild flavor attributes positively influenced hedonic scores, whereas stickiness, dryness, hardness, and to a lesser extent, visual aspect affected them negatively. As future perspectives, it could be interesting to involve a larger sample population to obtain more representative data about consumer responses to pulse snacks. Moreover, it could be useful to compare the present snacks with a savory or sweet filling and to involve a commercially available product type (e.g., rice-based snack with filling) to better understand the acceptance level of the prototype products

## Figures and Tables

**Table 1:** Hardness values of co-extruded snacks. Mean (*n* = 35) ± SEM. Different letters in the column correspond to significant differences (Tukey’s HSD test; *p* < 0.05).

Samples	Hardness (N)
R	48.3 ^cd^ ± 1.3
C	85.2 ^e^ ± 1.4
C15	47.4 ^c^ ± 0.8
C30	40.1 ^b^ ± 0.6
P	32.8 ^a^ ± 0.9
P15	46.4 ^c^ ± 0.9
P30	55.9 ^d^ ± 1.1

R = snacks from 100% rice; C = snacks from 100% chickpea; P = snacks from 100% green pea; C15 = snacks from 85% rice + 15% chickpea bran; C30 = snacks from 70% rice + 30% chickpea bran; P15 = snacks from 85% rice + 15% green pea bran; P30 = snacks from 70% rice + 30% green pea bran.

**Table 2:** Mean hedonic ratings ±SEM by samples, gender, and age groups. Hedonic scale range 0–100. Different letters show significant differences (*p* < 0.05) according to post hoc test.

Factors	Hedonic Ratings (Mean ± SEM)	F	*p*
Samples		5.58	**<0.0001**
R	42.4 ^a^ ± 1.9		
C30	49.2 ^b^ ± 1.6		
P30	49.8 ^b^ ± 1.5		
P15	51.5 ^bc^ ± 1.6		
C	53.0 ^bc^ ± 1.9		
C15	55.2 ^c^ ± 1.6		
P	56.0 ^c^ ± 2.3		
Gender		5.25	**0.02**
Females	49.3 ^a^ ± 0.9		
Males	52.7 ^b^ ± 1.1		
Age		5.95	**0.01**
≤26 years old	52.8 ^a^ ± 0.9		
>27 years old	49.2 ^b^ ± 1.0		

R = snacks from 100% rice; C = snacks from 100% chickpea; P = snacks from 100% green pea; C15 = snacks from 85% rice + 15% chickpea bran; C30 = snacks from 70% rice + 30% chickpea bran; P15 = snacks from 85% rice + 15% green pea bran; P30 = snacks from 70% rice + 30% green pea bran. Significant *p*-values are reported in bold

**Table 3:** Frequency counts (%) of check-all-that-apply (CATA) terms used to describe the extruded snacks and results of Cochran’s Q test for comparison among the samples.

Sensory Attributes	Samples						
	R	C30	P30	P15	C	C15	P
Appearance							
Dark yellow ***	1 ^a^	34 ^e^	25 ^cde^	12 ^abc^	18 ^bcd^	9 ^ab^	31 ^de^
Light yellow ***	58 ^c^	13 ^a^	12 ^a^	31 ^b^	40 ^b^	37 ^b^	12 ^a^
Green ***	0 ^a^	0 ^a^	0 ^a^	0 ^a^	0 ^a^	1 ^a^	24 ^b^
Odor							
Strong **	0 ^a^	2 ^ab^	4 ^ab^	2 ^ab^	6 ^ab^	1 ^a^	9 ^b^
Mild n.s.	32	31	30	27	28	37	27
Toasted ***	2 ^a^	20 ^b^	19 ^b^	17 ^b^	10 ^ab^	17 ^b^	9 ^ab^
Rice ***	16 ^c^	7 ^abc^	4 ^ab^	13 ^bc^	1 ^a^	14 ^bc^	4 ^ab^
Legume ***	3 ^a^	6 ^ab^	11 ^abc^	3 ^a^	18 ^c^	6 ^ab^	17 ^bc^
Flavor							
Mild ***	39 ^c^	28 ^bc^	34 ^bc^	38 ^c^	12 ^a^	38 ^c^	19 ^ab^
Strong ***	1 ^a^	9 ^a^	2 ^a^	2 ^a^	31 ^b^	0 ^a^	24 ^b^
Chickpea ***	5 ^a^	7 ^a^	5 ^a^	3 ^a^	31 ^b^	9 ^a^	13 ^a^
Rice ***	33 ^c^	12 ^ab^	24 ^bc^	27 ^bc^	5 ^a^	34 ^c^	5 ^a^
Pea ***	0 ^a^	5 ^a^	2 ^a^	3 ^a^	10 ^a^	0 ^a^	44 ^b^
Spicy ***	2 ^a^	6 ^a^	5 ^a^	7 ^a^	22 ^b^	1 ^a^	7 ^a^
Whole-wheat ***	2 ^a^	40 ^bc^	46 ^c^	27 ^b^	3 ^a^	39 ^bc^	4 ^a^
Taste							
Bitter n.s.	3	6	4	2	8	1	8
Salty n.s.	8	6	10	8	13	8	13
Sweet ***	10 ^ab^	27 ^c^	7 ^a^	21 ^bc^	8 ^a^	6 ^a^	12 ^ab^
Texture							
Crumbly ***	26 ^ab^	48 ^c^	34 ^bc^	46 ^c^	16 ^a^	43 ^c^	48 ^c^
Sticky ***	41 ^b^	15 ^a^	41 ^b^	32 ^b^	3 ^a^	37 ^b^	6 ^a^
Hard ***	54 ^b^	9 ^a^	13 ^a^	10 ^a^	62 ^b^	15 ^a^	14 ^a^
Porous ***	39 ^b^	37 ^b^	33 ^b^	38 ^b^	8 ^a^	41 ^b^	45 ^b^
Dry n.s.	54	44	49	43	47	44	40

R = snacks from 100% rice; C = snacks from 100% chickpea; P = snacks from 100% green pea; C15 = snacks from 85% rice + 15% chickpea bran; C30 = snacks from 70% rice + 30% chickpea bran; P15 = snacks from 85% rice + 15% green pea bran; P30 = snacks from 70% rice + 30% green pea bran. Different letters show significant differences (*p* < 0.05) according to post hoc test. N.s.= not significant; ** *p* < 0.01; *** *p* < 0.001.

**Table 4:** Pearson correlation coefficients among texture attribute perception and hardness measured by instrumental analysis (N).

	Hardness (N)	Crumbly	Hard	Porous	Sticky	Dry
Hardness (N)	1					
Crumbly	−0.87 *	1				
Hard	0.71 (*)	−0.92 **	1			
Porous	−0.96 **	0.79 *	−0.67	1		
Sticky	−0.20	0.04	−0.18	0.42	1	
Dry	0.39	−0.71 (*)	0.64	−0.26	0.53	1

(*) *p* < 0.10; * *p* < 0.05; ** *p* < 0.01.
